# Orthogonal analysis of variants in APOE gene using *in-silico* approaches reveals novel disrupting variants

**DOI:** 10.3389/fbinf.2023.1122559

**Published:** 2023-04-06

**Authors:** Chang Li, Ian Hou, Mingjia Ma, Grace Wang, Yongsheng Bai, Xiaoming Liu

**Affiliations:** ^1^ USF Genomics and College of Public Health, University of South Florida, Tampa, FL, United States; ^2^ The John Cooper School, The Woodlands, TX, United States; ^3^ Novi High School, Novi, MI, United States; ^4^ Del Norte High School, San Diego, CA, United States; ^5^ Next-Gen Intelligent Science Training, Ann Arbor, MI, United States; ^6^ Department of Biology, Eastern Michigan University, Ypsilanti, MI, United States

**Keywords:** AlphaFold, missense variant, APOE, Alzheimer’s disease, deep learning, ensemble

## Abstract

**Introduction:** Alzheimer’s disease (AD) is one of the most prominent medical conditions in the world. Understanding the genetic component of the disease can greatly advance our knowledge regarding its progression, treatment and prognosis. Single amino-acid variants (SAVs) in the APOE gene have been widely investigated as a risk factor for AD Studies, including genome-wide association studies, meta-analysis based studies, and *in-vivo* animal studies, were carried out to investigate the functional importance and pathogenesis potential of APOE SAVs. However, given the high cost of such large-scale or experimental studies, there are only a handful of variants being reported that have definite explanations. The recent development of *in-silico* analytical approaches, especially large-scale deep learning models, has opened new opportunities for us to probe the structural and functional importance of APOE variants extensively.

**Method:** In this study, we are taking an ensemble approach that simultaneously uses large-scale protein sequence-based models, including Evolutionary Scale Model and AlphaFold, together with a few *in-silico* functional prediction web services to investigate the known and possibly disease-causing SAVs in APOE and evaluate their likelihood of being functional and structurally disruptive.

**Results:** As a result, using an ensemble approach with little to no prior field-specific knowledge, we reported 5 SAVs in APOE gene to be potentially disruptive, one of which (C112R) was classificed by previous studies as a key risk factor for AD.

**Discussion:** Our study provided a novel framework to analyze and prioritize the functional and structural importance of SAVs for future experimental and functional validation.

## 1 Introduction

Alzheimer’s disease (AD), a complex disease with a known genetic basis, is the most prominent cause of dementia in the elderly (Bettens et al., 2013). Understanding the genetic component of AD can be of great importance in its early diagnosis, effective treatment and improved prognosis. It has been widely studied and reported that the apolipoprotein E (APOE) gene, which is a key gene for lipid transportation, is closely associated with the risk of AD (Kamboh et al., 1995; Yamazaki et al., 2019; Martens et al., 2022). APOE gene has 3 different protein isoforms, namely, APOE2, APOE3, and APOE4 (Husain et al., 2021). These isoforms differ by two amino acids, APOE2 with Cys112 and Cys158, APOE3 with Cys112 and Arg158, and APOE4 with Arg112 and Arg158. APOE3 was considered the reference isoform and the APOE4 Cys112Arg variant was a strong risk factor for AD, while APOE2 Arg158Cys variant was reported to be protective (Bojanowski et al., 2006; Dolai et al., 2020). Given the functional importance and pathogenesis potential of APOE variants, many experimental studies using animal models, genome-wide association studies, and other meta-analyses have been performed to interrogate the impact of variants residing in the APOE gene (Bertram et al., 2008; Liu et al., 2014; Lewandowski et al., 2020). However, given the high cost of such large-scale or experimental studies, there are only a handful of variants being reported that have definite explanations.

The recent development of *in silico* analytical approaches, especially large-scale deep learning models, has opened new opportunities for us to probe the structural and functional importance of APOE variants extensively. Specifically, AlphaFold (Jumper et al., 2021), which exploited attention mechanisms from language modeling and multiple sequence alignment (MSA) data of protein homologs, has provided substantially increased coverage of high-confidence protein structure predictions. Additionally, the Evolutionary Scale Model (ESM) (Lin et al., 2022), which was pre-trained on 250 million protein sequences, has proven to be able to extract key functional domains and evaluate the functional importance of amino acid variants (Brandes et al., 2022) even in the absence of multiple sequence alignment (MSA) data which were required in AlphaFold modeling. Recent studies have tried to examine the ability of these tools individually to evaluate the impact of single amino-acid variants (SAVs), but reported conflicting results (Pak et al., 2021; Caswell et al., 2022). In this study, instead of using these tools separately, we are taking an ensemble approach that simultaneously uses these two large-scale protein sequence-based models together with a few *in silico* functional prediction web services to investigate the known and possibly disease-causing variants in APOE and evaluate their likelihood of being functional and structurally disruptive.

## 2 Materials and methods

### 2.1 APOE sequence data retrieval

The protein sequence of the APOE gene was retrieved from Ensembl genome browser v107 in FASTA format (https://useast.ensembl.org/index.html). Python package Biopython was used to load and process the retrieved sequence. Only the reference isoform (APOE3) and the precursor APOE (pre-APOE) sequences were used in this study. The difference between pre-APOE and mature APOE was the addition of an 18-residue signal peptide at the beginning of the sequence. As a result, previously reported variants with respect to mature APOE, such as C112R and R158C, were reported as C130R and R176C, respectively, in this study.

The C130R variant was manually introduced to create a separate sequence representing APOE4, and R176C was manually introduced to create a separate sequence representing APOE2.

### 2.2 ESM model retrieval and variant effect prediction

ESM-1b model was retrieved from GitHub (https://github.com/facebookresearch/esm) using PyTorch Hub. The same tokenizer as the original ESM model was used to encode input protein sequences. The variant effect for each amino acid variant (ESM score) was calculated as the log-likelihood ratio between the variant and the corresponding reference amino acid. To show a positive score, we multiplied each prediction score by −1.

$$ESM\;score=-\log\frac{P\left(Variant\right)}{P\left(Reference\right)}$$

The variant was predicted to be more damaging if it had a higher ESM score.

### 2.3 AlphaFold model retrieval and variant effect prediction

AlphaFold v2 model was run locally using a third-party implementation, namely, LocalColabFold (https://github.com/YoshitakaMo/localcolabfold) (Jumper et al., 2021; Mirdita et al., 2022). The algorithm first implements MMseqs2 (Steinegger and Söding, 2017) to retrieve MSA for the target protein. Then, it predicts the 3D protein conformation for the given sequence.

Due to the high computational cost of running AlphaFold, it was extremely time-consuming to run predictions (*in silico* mutagenesis) for all possible SAVs in APOE, which would require running AlphaFold 6,023 times (Supplementary Table S1). As a workaround, we retrieved all SAVs in APOE reported in ClinVar (Landrum et al., 2015). First, ClinVar database version 20220507 was downloaded from https://ftp.ncbi.nlm.nih.gov/pub/clinvar/. Second, only variants annotated as inside the APOE gene were kept (*n* = 69). Third, only non-synonymous single nucleotide variants were kept, and all insertions and deletions were excluded (*n* = 38). As a result, a total of 38 SAVs were retrieved, and a separate protein sequence was created for each SAV. The predicted 3D protein structure for the wild-type and each mutant sequence was compared using the root-mean-square deviation (RMSD) of atomic positions, which was commonly used as a distance measurement between two protein structures. A variant with a higher RMSD score was expected to have a greater impact on the protein structure. Therefore, the RMSD score was used as a surrogate for AlphaFold’s prediction of the variant’s impact.

### 2.4 Missense3D and DynaMut2 web service tools

Besides the two computational tools described previously, we used two additional web services to measure/predict the stability of the protein with and without the variants. First, the Missense3D database for APOE was retrieved from http://missense3d.bc.ic.ac.uk:8080 (Khanna et al., 2021), which contains 307 pre-calculated predictions in APOE. Second, DynaMut2 was used to predict user supply variants (Rodrigues et al., 2020). The same SAVs retrieved from ClinVar were used and submitted to the DynaMut2 web service at: https://biosig.lab.uq.edu.au/dynamut2/.

### 2.5 Retrieval of additional annotations

To evaluate the performance of the main predictor (ESM-1b model), we retrieved population allele frequencies from gnomAD (https://gnomad.broadinstitute.org/news/2020-10-gnomad-v3-1/). Maximum population frequencies were retrieved for the same SAVs retrieved from ClinVar as described previously.

An Evolutionary conservation score, GERP++ (Davydov et al., 2010), was retrieved from the dbNSFP v4.3a database (Liu et al., 2011; Liu et al., 2020), available at https://sites.google.com/site/jpopgen/dbNSFP.

Additionally, we have retrieved 3 popular tools for predicting protein stability change upon mutation, namely, FoldX (Schymkowitz et al., 2005), DDGun (Montanucci et al., 2019) and Maestro (Laimer et al., 2015). First, FoldX was downloaded from https://foldxsuite.crg.eu/ using the academic license. The “Stability” command was used to calculate the Gibbs energy of protein folding for all 38 potential SAVs. The difference in folding energy between wild-type and mutant sequences was calculated and their absolute values were used to represent each SAV’s impact predicted by FoldX, since both stabilizing and destabilizing mutations may all have substantial impacts on the function of the protein. Second, the DDGun web service, available at: https://folding.biofold.org/ddgun/index.html, was used to make predictions on protein stability change given a list of mutations. Specifically, the wild-type sequence of APOE with a list of IDs for all 38 SAVs was uploaded. A global Delta Delta G (DDG) value was predicted for each of the SAVs, and its absolute value was used to represent each SAV’s impact predicted by DDGun. Third, Maestro v1.2.35 Linux executable file was downloaded from https://pbwww.services.came.sbg.ac.at/?page_id=477. All 38 SAVs were submitted as input for the Maestro program with the wild-type 3D structure of APOE obtained using AlphaFold2. Similarly, the DDG values were obtained from the prediction and their absolute values were used to represent each SAV’s impact predicted by Maestro.

### 2.6 Statistical tests and visualizations

To evaluate the correlation between allele frequencies of the variants and predictions made by computational tools, Pearson’s correlation coefficient was calculated using the Python library SciPy (https://scipy.org/). We calculated the area under the receiver operating characteristic curve (auROC) and average precision scores to evaluate each predictor’s ability in prioritizing potential clinically relevant variants. Specifically, an auROC was calculated by measuring the predictor’s true positive rate (TPR) and false positive rate (FPR) using different score cutoffs. Similarly, the average precision (AP) score was calculated by measuring the predictor’s precision and recall (same as TPR) using different score cutoffs. The formulas for calculating TPR, FPR, and precision are:

$$TPR\;\left(Recall\right)=\frac{TP}{TP+FN}$$

$$FPR=\frac{FP}{TN+FP}$$

$$Precision=\frac{TP}{TP+FP}$$

Where TP refers to the number of true positives (correctly predicted ClinVar pathogenic variants), FN refers to the number of false negatives (incorrectly predicted ClinVar pathogenic variants as benign), FP refers to the number of false positives (incorrectly predicted ClinVar benign variants as pathogenic), and TN refers to the number of true negatives (correctly predicted ClinVar benign variants). Both auROC and AP scores were calculated using the Python library sklearn with functions *roc_auc_score* and *average_precision_score*, respectively.

Additionally, PyTorch was used to calculate ESM model predictions, and Tensorflow was used to calculate AlphaFold model predictions.

## 3 Results

### 3.1 ESM-1b model can predict regions with high importance

**FIGURE 1:**
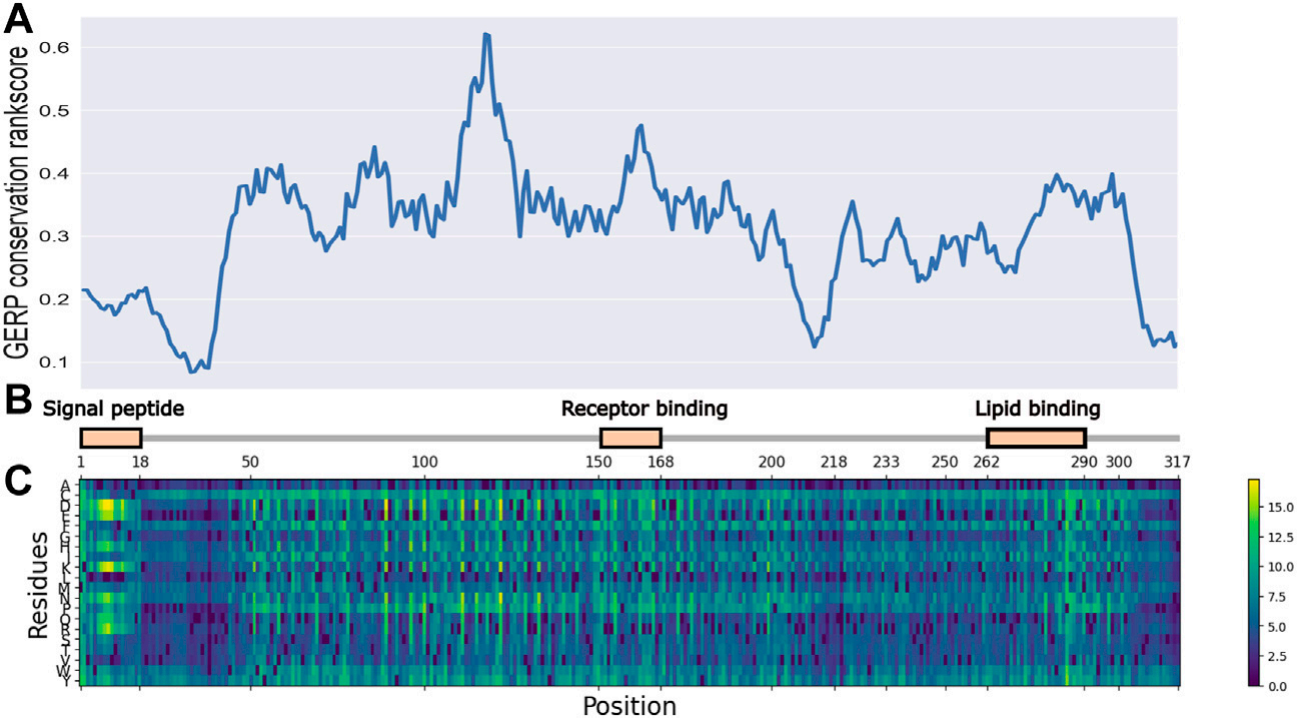
ESM-1b and GERP predicted functional importance scores for all potential SAVs in APOE gene. **(A)** GERP conservation scores for APOE gene. **(B)** functional domains for APOE gene. **(C)** ESM-1b *in silico* mutagenesis predictions for APOE gene.

As illustrated in Figure 1, the entire length of the APOE protein was predicted by the ESM-1b model, and all potential amino acid variants were evaluated as the log odds ratio between the mutant and wild-type predictions. Variants with lighter colors indicate a low predicted likelihood of the existence of a variant at this position, which implies their functional importance. Key functional domains, including a signal peptide, receptor binding domain and lipid binding domain showed higher importance, as illustrated by light color bands. Interestingly, these regions of high importance showed higher conservation scores (GERP++ score), as illustrated by the top panel. In contrast, amino acids from positions 18–45 showed both low conservation and low predicted functional importance. This observed concordance of the ESM prediction with annotated functional domains and evolutionary conservation demonstrated the model’s ability to capture important regions in the APOE gene, given that the gene is only moderately conserved and is quite tolerant to missense variants (Lek et al., 2016).

Additionally, multiple clustering patterns were observed in the prediction heatmap, as illustrated by regions with high predicted values. One of these regions was amino acids 1–18, representing the signal peptide region. While few studies have tried to evaluate the functional importance of variants residing in this region, it is clear that multiple variants can be extremely harmful to the protein’s function.

### 3.2 ESM-1b model can identify variants of high functional importance in the population

**FIGURE 2:**
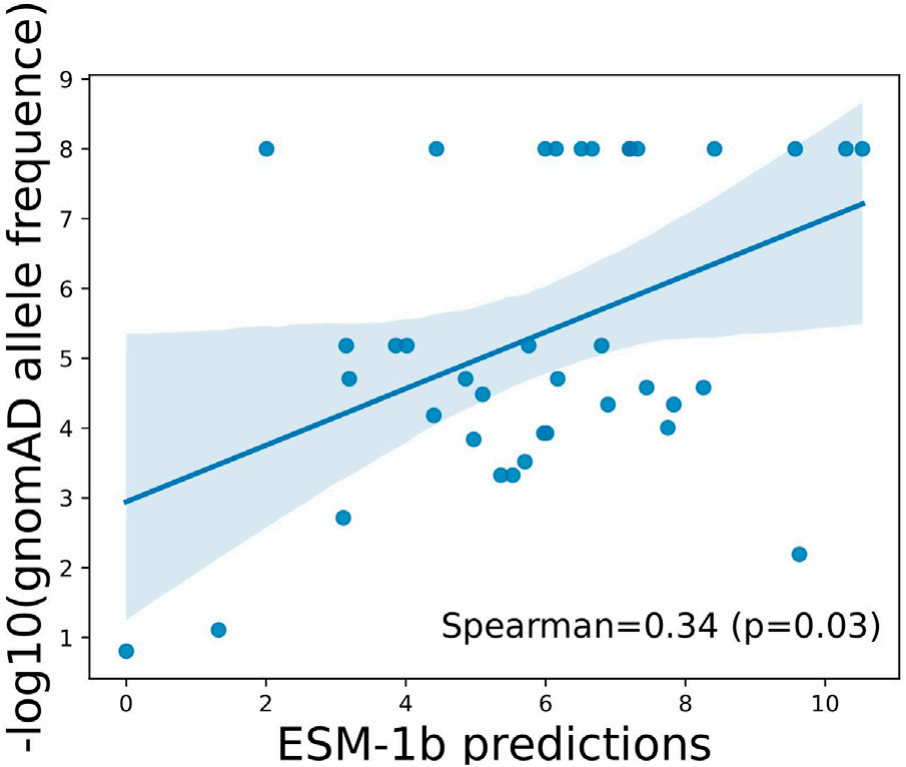
Correlation between ESM-1b predictions and population allele frequencies among ClinVar reported variants in APOE. Pearson correlation coefficient and associated *p*-value were reported.

To illustrate if the scores predicted by ESM-1b can truly reflect function importance at the variant level, we next evaluated allele frequencies observed in a large-scale population cohort, namely, gnomAD, and see if the model’s predictions show correlations with allele frequencies (AFs) of the variants in general populations. Due to purifying selection, variants with lower AFs are more likely to be deleterious, whereas variants with higher AFs are more likely to be tolerated (benign). As illustrated in Figure 2, predictions by ESM-1 showed statistically significant positive correlations with −log_10_(AFs) in the general population, which indicates its capability of identifying truly functional variants that have undergone purifying selection.

### 3.3 AlphaFold’s predictions correlate with evolutionary conservation

**FIGURE 3:**
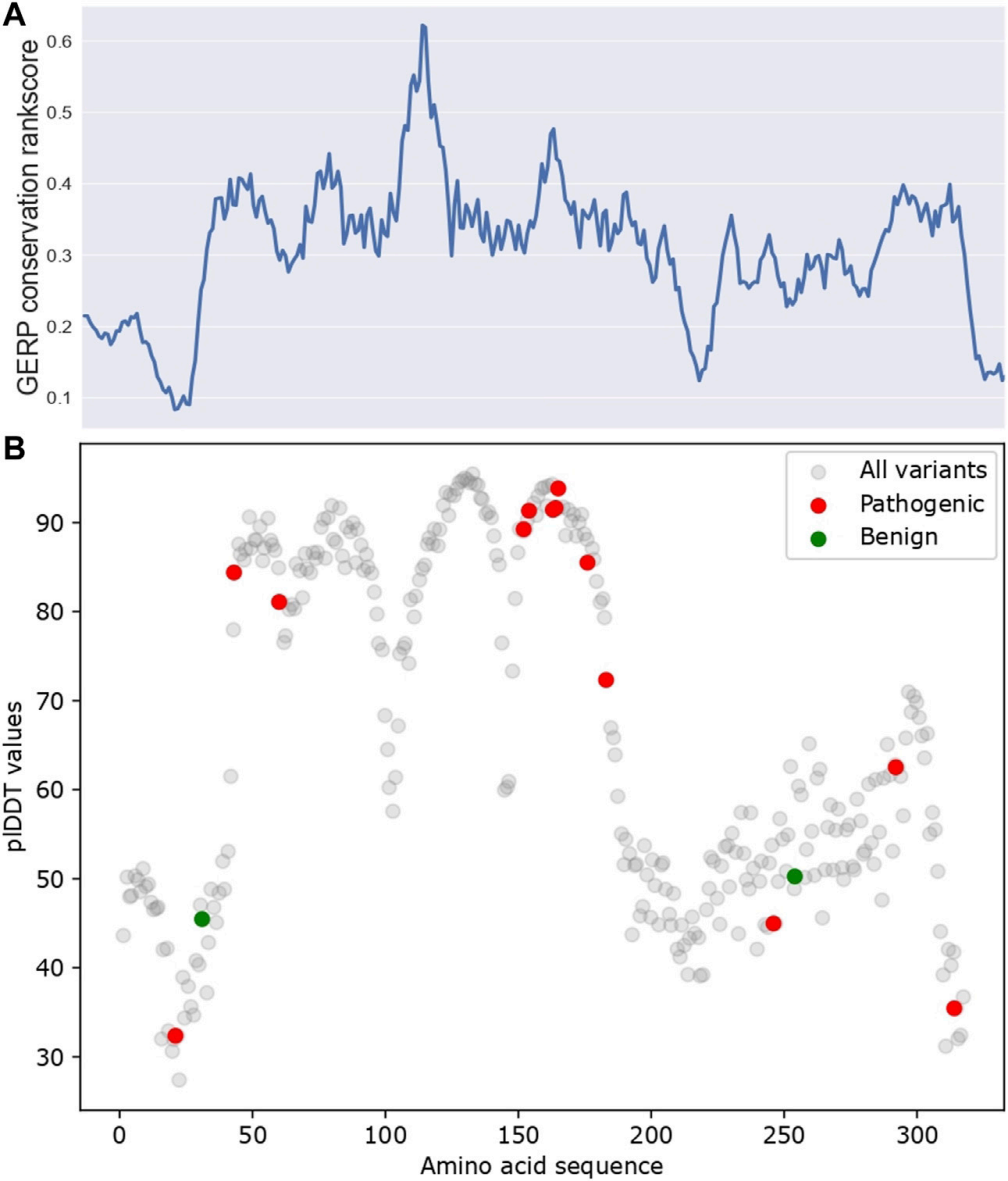
The pLDDT scores for AlphaFold predicted APOE structure. **(A)** GERP conservation score. **(B)** pLDDT scores along APOE protein sequence. ClinVar pathogenic/benign variants were highlighted in red and green, respectively. All variants are referred to as all amino acids in APOE, which reflect the pLDDT distribution for all amino acids of APOE.

Next, we investigated the predictions made by AlphaFold. AlphaFold gives a per-residue confidence metric called pLDDT (predicted Local Distance Difference Test) score for all alpha-carbon atoms (Mariani et al., 2013). Regions with high pLDDT scores usually have fewer clashes and structural violations. As shown in Figure 3, pLDDT scores correlate with conservation scores (Spearman correlation coefficient = 0.43, *p*-value = 1.94 × 10^−15^), which was expected, as AlphaFold prediction relies on MSA data as input, which primarily utilizes conservation data. Additionally, in regions with high pLDDT scores (pLDDT >70), for example, the amino acid’s approximate position from 45–170, only pathogenic variants and no benign variants were reported. Their potential structural importance could explain this observed pattern.

### 3.4 Orthogonal tools show low pairwise correlations

**FIGURE 4:**
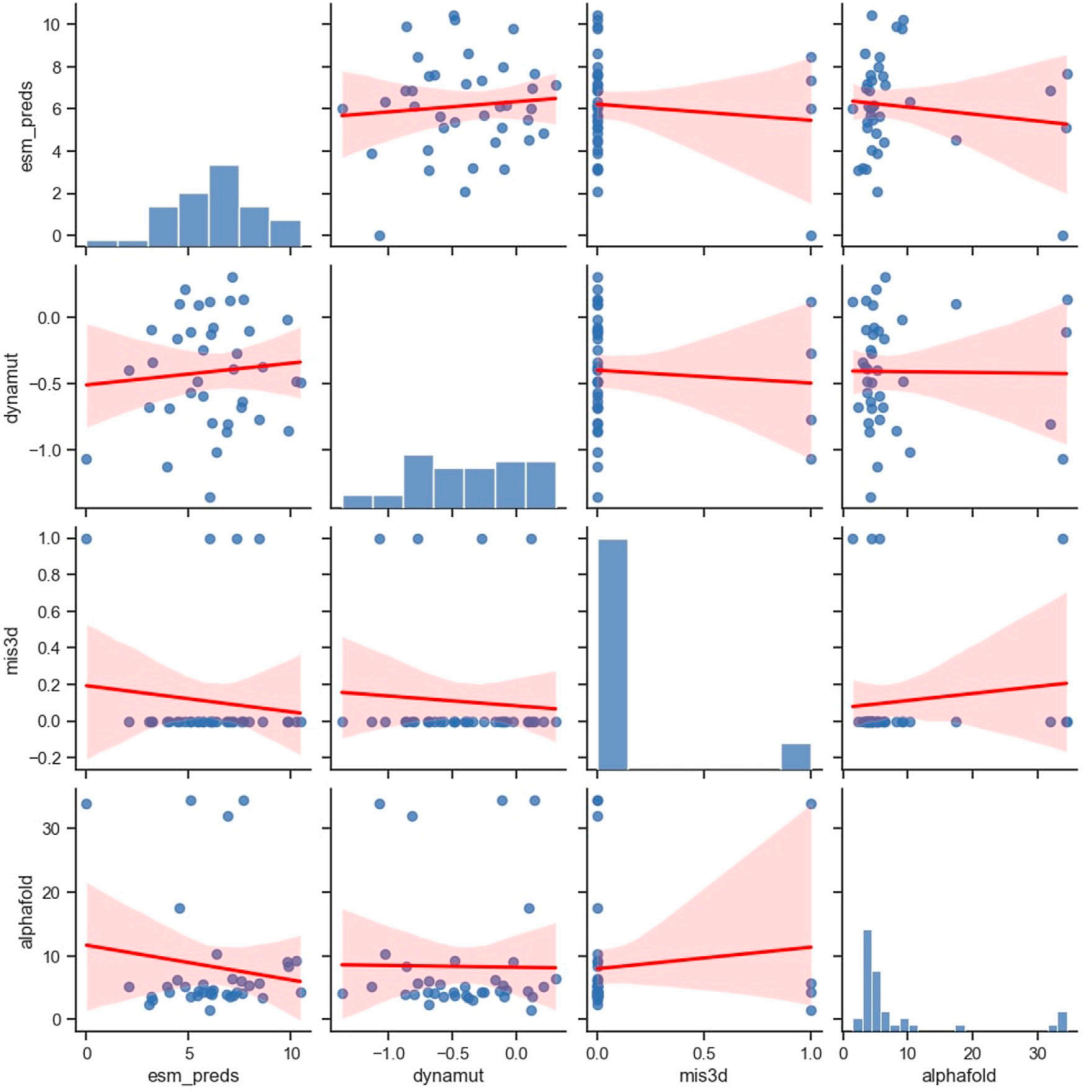
Pairwise correlation of the variants between the four *in silico* predictors.

We compared the correlation of the predictions made by four popular computational frameworks, namely, ESM-1b, AlphaFold, Missense3D, and DynaMut2, which measure protein properties from different perspectives (Figure 4). Specifically, ESM model studies comprehensive protein sequence features from millions of protein sequences using a language model. AlphaFold model studies protein sequences and tries to predict 3D protein structures using sequences and available templates. Missense3D adopts a bioinformatics pipeline and evaluates a wide range of structural impacts of an SAV. DynaMut2 model predicts protein stability by learning a series of biochemical and biophysical features from the target proteins. We have examined additional popular *in silico* tools that can predict protein stability (Caldararu et al., 2020; Pan et al., 2022), including FoldX (Schymkowitz et al., 2005), DDGun (Montanucci et al., 2019) and Maestro (Laimer et al., 2015), but all of them showed inferior performance compared to DynaMut2 in APOE (Supplementary Figures S1, S2; Supplementary Table S2). Therefore, we chose only DynaMut2 as the representative tool for protein stability prediction. Interestingly, benchmarked using ClinVar labels, the DynaMut2, as the best individual predictor among protein stability methods, outperformed predictors from other categories, including ESM and AlphaFold. Therefore, we provided predictions from DynaMut2 for all possible SAVs in APOE in Supplementary Table S3.

All these four selected tools showed no or very weak correlations with each other, which can be both concerning and useful. On one hand, if the inconsistency arises from methodological flaws, their ability to capture useful information is extremely limited. Users should be cautious when adopting these tools in their workflow. On the other hand, if this inconsistency arises from the differences in methodological preferences and their ability to capture different aspects of protein functions, then these tools can provide valuable orthogonal information.

### 3.5 Ensemble of multiple tools can provide biological meaningful insights

**FIGURE 5:**
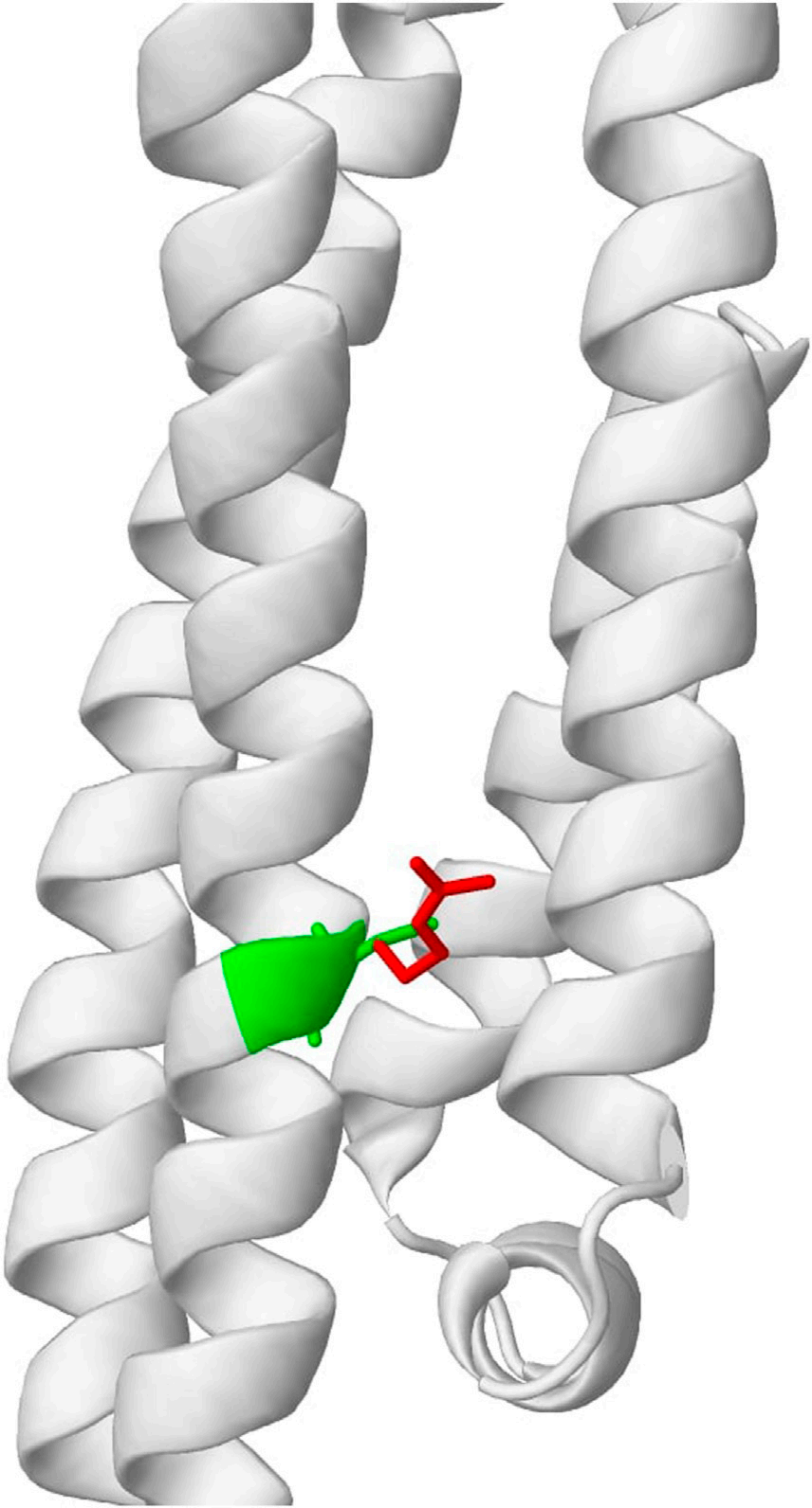
Predicted change in residue properties between Cysteine (green) and Arginine (red) in C130R variant by Missense3D.

To evaluate the usefulness of the previously described tools and illustrate if their low correlation can be beneficial to explaining variant effects, we obtained top candidates from multiple predictions and examined their biological relevance as a means of validation. The top candidates were obtained based on the predictions made by each of the four tools. We consider variants to be potentially pathogenic if predictions from two or more tools showed indicative of a disruptive effect. Using this ensemble approach (majority vote), five candidate variants were obtained. As shown in Table 1, variants C130R, R163C, and R132C are most likely to be functional. Importantly, DynaMut2, which predicts the stability of the mutant protein sequence, showed destabilizing effects for all these 3 variants. AlphaFold models also predict the top 2 variants to disrupt the key functional domains. Interestingly, the most promising variant, C130R, is the variant that separates the transcript that carries the variant gene (APOE4) from the wild-type transcript (APOE3). The variant replaces Cysteine with Arginine, which was predicted to change a residue state from buried to exposed (Figure 5). The functional importance of the C130R variant was validated by previous studies, which reported the variant to be associated with an elevated risk of AD (Husain et al., 2021; Martens et al., 2022). This observation highlighted the ability to combine multiple functional prediction tools in finding key functional variants.

**TABLE 1:** Five candidate variants that affect APOE function.

Variant	Allele frequency	ESM	DynaMut	Missense3D	AlphaFold	Evidence count
C130R	0.138	0	**−1.07**	**1**	**33.813**	3
R163C	0.001	**9.631**	**−0.86**	0	**8.305**	3
R132C	0.00003	**8.259**	**−0.77**	**1**	5.664	3
R163P	NA	**10.296**	−0.48	0	**9.320**	2
R160C	NA	**9.571**	−0.02	0	**9.113**	2

*ESM, cutoff = 7.97(top 25 percentile); DynaMut cutoff = −0.5; Missense3D = 1 (damaging); AlphaFold cutoff = 6.42 (top 25 percentile). *specifies which cutoff value was used for each of these predictors to decide if their predictions for the variants are damaging (functional) or not (non-functional).

The bold values mean that the individual predictor’s prediction for that specific variant is damaging (functional).

Next, using a similar approach, we identify one variant to be potentially benign. As shown in Table 2, all four tools predicted the variant to be non-functional. ClinVar reported a conflicting interpretation of pathogenicity for this SAV, meaning that multiple clinical laboratories reported contradictory interpretations for the same variant. Specifically, some studies reported it to be benign while others report it to be uncertain significance, according to the 2015 ACMG-AMP guidelines (Richards et al., 2015). Given its previous uncertain annotations and the fact that all orthogonal *in silico* methods showed concordant prediction, its function is worth investigating in future studies to confirm whether the SAV is truly benign. All calculated scores for all 38 SAVs analyzed in the study are provided in Supplementary Table S2.

**TABLE 2:** Candidate variant that predicted to be benign by all tools.

Variant	Allele frequency	ESM	DynaMut	Missense3D	AlaphaFold
L46P	0.0025	3.106	−0.09	0	3.564

## 4 Discussion

In this study, we explored the usefulness of various orthogonal *in silico* predictors in their ability to prioritize functionally and structurally disruptive SAVs in the APOE gene. Using little to no prior knowledge, we identified 5 potentially disrupting variants, one of which (C130R) was classified by previous studies as a key risk factor for AD (Holtzman et al., 2012).

As illustrated by our study, the ESM model, which utilized large-scale pretraining and state-of-the-art deep learning architectures, can efficiently identify highly important domains and functional SAVs. The N-terminal of the APOE protein consists of 4 helices, H1, H2, H3, and H4, which form a four-helix bundle that spans amino acids from 42 to 182 (Wilson et al., 1991). These helices contain some key functional domains, such as the LDL-receptor binding region (residues 154–168). As illustrated by the ESM prediction (Figure 1), this region indeed contains multiple highlighted bands, reflecting the potential functional importance of the variants. Moreover, the previously mentioned domain for the signal peptide (residues 1–18) represents another region of interest. It has been previously reported that variants located in signal-peptide-encoding sequences may severely impact protein transportation (Jarjanazi et al., 2007). For this under-investigated region, no variant was reported in ClinVar, including benign, pathogenic, or variant of unknown significance (VUS), which calls for future studies to perform functional validation of variants in this region that focus on the transportation and maturation of APOE.

However, the ESM model was imperfect, and it may fail to predict variants residing in regions with little homologous coverage. For example, in our study, the ESM model incorrectly predicted the C130R variant to be non-functional. On the other hand, the AlphaFold model has demonstrated potential in identifying such highly disruptive SAVs. While the C130R variant was predicted as non-functional by the ESM model, it showed the highest disruptive effect predicted by AlphaFold among the top 5 candidate SAVs. Based on our results from non-specific *in silico* predictions, this C130R variant may convey its functional impact through altered protein 3D structure rather than the function encoded in the underlying amino acid. Indeed, this C130R, or the equivalently C112R in mature APOE, was reported to destabilize the protein structure, which was considered to improve its ability to bind to lipid and amyloid-β surfaces, which may ultimately increase the risk of AD (Chetty et al., 2017).

Aside from the promising results of using a set of orthogonal *in silico* tools to help us understand the functional importance of APOE variants, we believe there are a few limitations in our study that future studies could improve upon. First, our illustration and analysis in this study were based only on a single gene APOE, and future studies may include other apolipoprotein genes to investigate the capability of these novel computational tools in assisting lipid research. Second, we only considered SAVs in this study, and we note that InDels (short insertions or deletions) may play a greater role in protein stability and function. It is still an open question regarding if and how these existing computational tools can help with this regard. Third, in this study, we performed validation across multiple data resources, including conservation score and population allele frequency, and future studies may be conducted to include additional *in silico* validations and even experimental validations, such as deep mutational scanning data, to further elucidate the functional importance of the reported variants.

## Data Availability

The original contributions presented in the study are included in the article/Supplementary Material, further inquiries can be directed to the corresponding authors.

## Supplementary material

SUPPLEMENTARY FIGURE S1 Performances of all quantitative predictors analyzed in this study benchmarked using ClinVar labels. The dashed red line represents the ROC cut-off value. Tools with greater ROC values were chosen to construct the ensemble.

SUPPLEMENTARY FIGURE S2 ROC curves for all quantitative predictors analyzed in this study benchmarked using ClinVar labels.
