# Supplementary figures and images for: Orthogonal analysis of variants in APOE gene using in-silico approaches reveals novel disrupting variants
Source: Front Bioinform. 2023 Apr 6;3:1122559. doi: 10.3389/fbinf.2023.1122559 (PMC10117898; doi:10.3389/fbinf.2023.1122559)

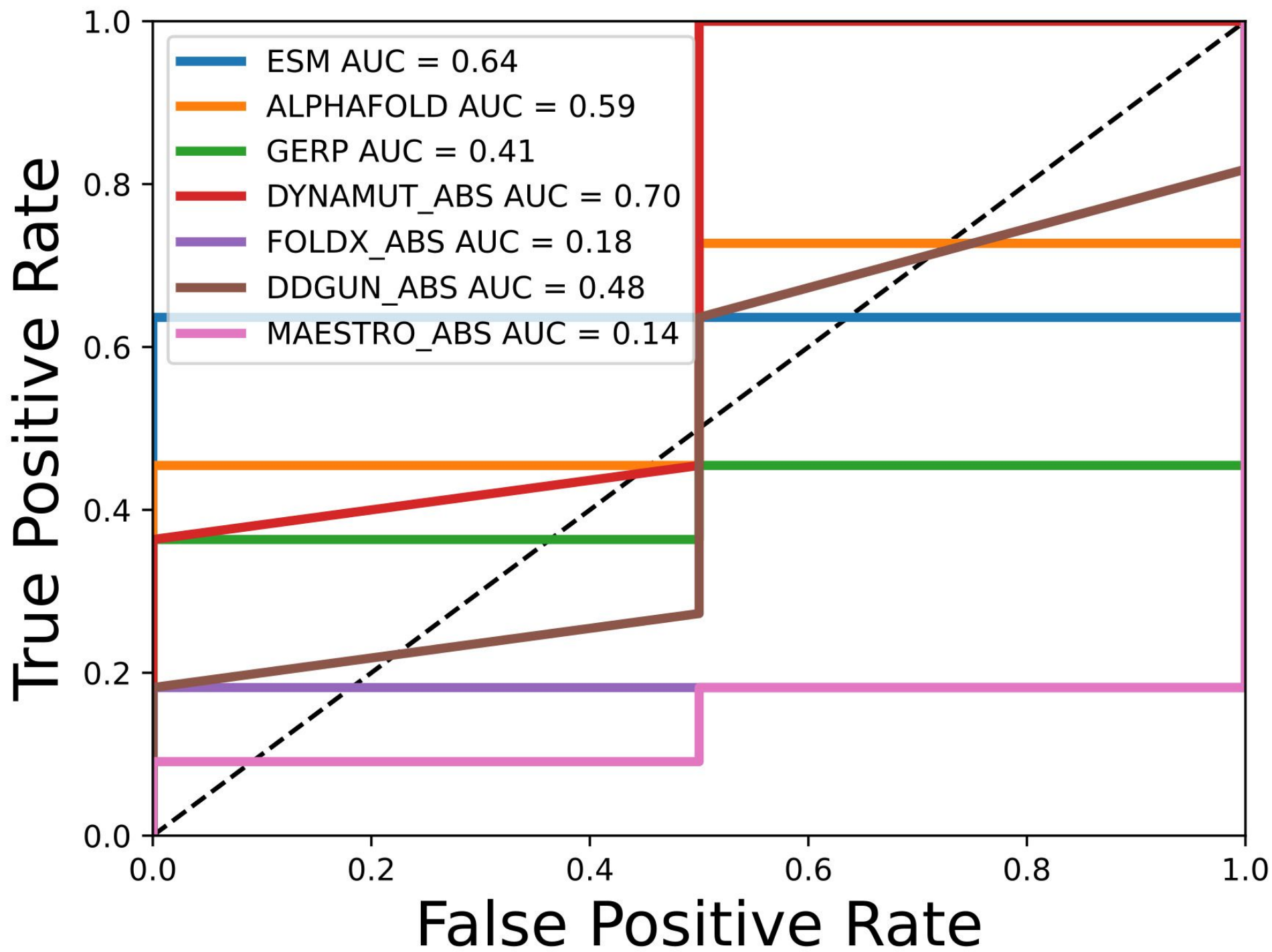

Supplement: Supplementary file 2 [file Image2.PDF]

Average Precision

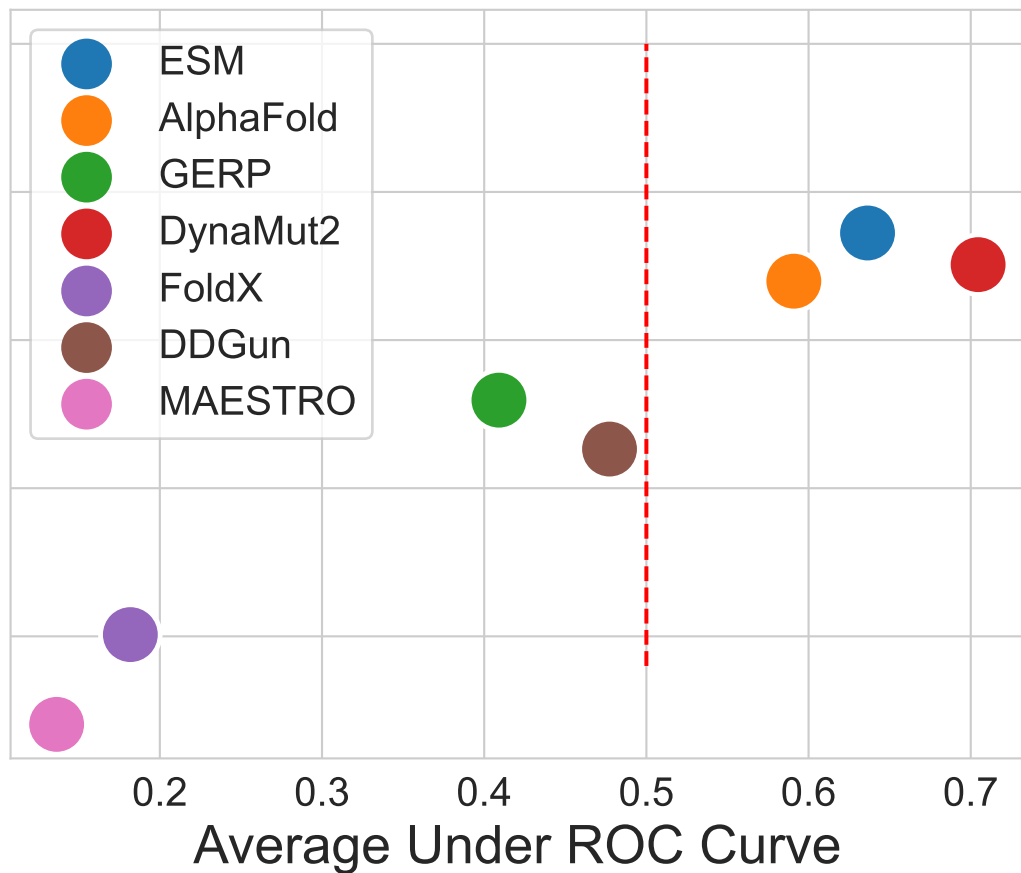

Supplement: Supplementary file 5 [file Image1.PDF]
